# Case Report: Does extracorporeal membrane oxygenation treatment for acute pulmonary embolism-induced respiratory and cardiac arrest still require thrombolysis?

**DOI:** 10.3389/fcvm.2025.1578970

**Published:** 2025-05-22

**Authors:** Fangfang Qiu, Bingxin Song, Lina Chen, Jiayi Hong

**Affiliations:** ^1^ Department of Critical Care Medicine, the Fourth Affiliated Hospital of School of Medicine, and International School of Medicine, International Institutes of Medicine, Zhejiang University, Yiwu, China; ^2^ Department of Respiratory and Critical Care Medicine, Center for Oncology Medicine, the Fourth Affiliated Hospital of School of Medicine and International School of Medicine, International Institutes of Medicine, Zhejiang University, Yiwu, China

**Keywords:** pulmonary embolism—diagnosis, extracorporeal membrane oxygenation (ECMO), thrombolysis, cardiac arrest (CA), disseminated intravascular coagulation (DIC)

## Abstract

Acute massive pulmonary embolism (PE) secondary to cardiac arrest (CA) is associated with extremely high mortality. Venoarterial extracorporeal membrane oxygenation (VA-ECMO) serves as a critical life support modality; however, the safety and necessity of combined thrombolytic therapy remain controversial. This study reports the clinical outcomes of two CA patients with acute PE treated with VA-ECMO: Case 1 underwent ECMO support without thrombolysis, receiving only heparin anticoagulation. Dynamic imaging evaluation demonstrated gradual thrombus resolution, leading to successful weaning from ECMO and subsequent recovery. Case 2 received immediate thrombolysis with alteplase 50 mg after ECMO cannulation but succumbed to severe bleeding complications—including cannulation site hemorrhage, disseminated intravascular coagulation (DIC), and hemorrhagic shock—within 24 h. For ECMO-treated PE patients with CA, clinical decisions should be based on etiological assessment, bleeding risk, and multimodal evaluations (e.g., imaging, coagulation function), prioritizing individualized reperfusion strategies (such as catheter-directed thrombectomy or surgical embolectomy) to improve prognosis. Although both cases described herein received VA-ECMO as salvage therapy, their divergent thrombolytic strategies resulted in contrasting clinical outcomes, prompting critical clinical reflections on risk-benefit balancing in this high-risk population.

## Introduction

Pulmonary embolism (PE) is a life-threatening critical illness with a mortality rate as high as 30%–50%, while the mortality in patients with secondary cardiac arrest (CA) escalates to 84%–95% (1, 2). Extracorporeal membrane oxygenation (ECMO), as an effective mechanical circulatory support modality, provides transitional therapy for patients with severe cardiopulmonary dysfunction, improving systemic circulatory perfusion and gaining time for etiological diagnosis and intervention (1). For PE-induced CA patients, venoarterial extracorporeal membrane oxygenation (VA-ECMO) can rapidly stabilize hemodynamics; however, controversy remains regarding the adjuvant use of thrombolytic therapy. Thrombolytic therapy is a first-line recommended strategy for high-risk PE, but its safety and necessity under ECMO support remain undefined (3). ECMO circuits inherently require anticoagulation management, and thrombolysis may exacerbate bleeding risk—studies have shown that 60% of ECMO-related complications are associated with bleeding, directly linked to in-hospital mortality (1). Although European guidelines suggest considering thrombolysis for hemodynamically unstable high-risk PE patients, ECMO application may alter clinical decision-making: on the one hand, ECMO extends the time window for thrombolysis; on the other hand, thrombolysis may trigger life-threatening bleeding [e.g., cannulation site hemorrhage and disseminated intravascular coagulation (DIC)], with significantly higher risks in postoperative or pre-existing anticoagulated states (2, 3). Currently, data on the efficacy and safety of thrombolysis under ECMO support in PE patients with CA are limited, and substantial heterogeneity exists in clinical practice. By analyzing two cases of acute PE-induced CA treated with VA-ECMO, comparing their thrombolytic strategies and clinical outcomes, and integrating a literature review, this paper explores the optimal reperfusion strategies for high-risk PE in the ECMO era, aiming to provide a clinical reference for balancing thrombolytic and bleeding risks.

## Case 1

**Figure 1:**
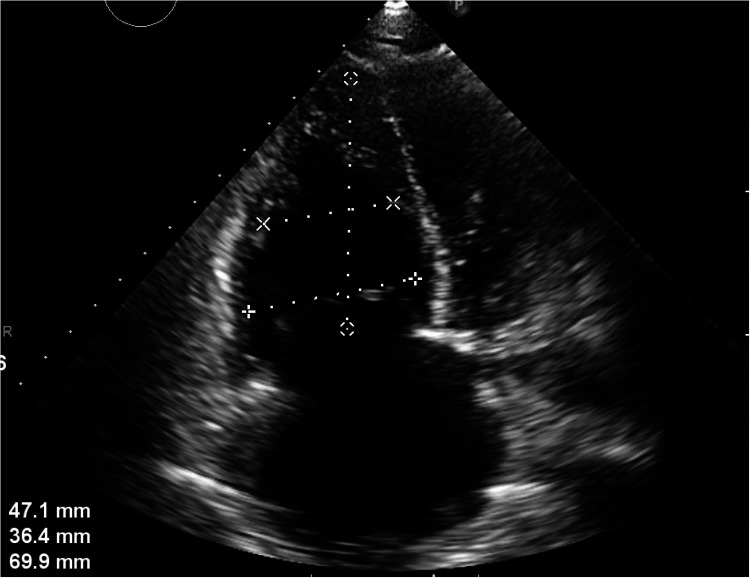
Apical four-chamber section: the right heart is significantly enlarged and compresses the left heart, showing a “D-sign.” The right ventricular end-diastolic diameter is approximately 47.1 × 69.9 mm.

**Figure 2:**
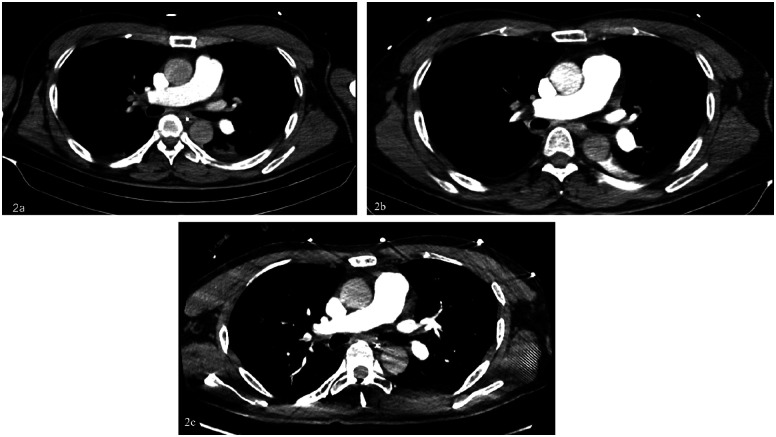
**(A)** Day 0: multiple emboli in the right pulmonary artery trunk and both pulmonary artery branches, exudative changes in both lungs, and a small amount of pericardial effusion. **(B)** Day 3: multiple emboli in the right pulmonary artery and branches of both pulmonary arteries, slightly absorbed compared to the anterior part. **(C)** Day 13: the embolism in the right pulmonary artery trunk was significantly absorbed compared with before, and the contrast agent was well filled.

The patient is a 56-year-old factory assembly line worker, with previous good health, who presented to a local hospital with symptoms of chest tightness and dizziness. An initial electrocardiogram (ECG) showed no significant abnormalities, and the D-dimer level was 4.25 mg/L. The patient was diagnosed with a transient ischemic attack (TIA) and admitted to the neurology department for treatment. Five hours later, the patient experienced a sudden syncope, followed by cardiac arrest (CA). After approximately 40 min of cardiopulmonary resuscitation (CPR), a return of spontaneous circulation (ROSC) was achieved. However, 10 min later, the patient experienced another episode of CA, requiring another 5 min of CPR to restore ROSC. Bedside cardiac ultrasound revealed significant right heart enlargement compressing the left heart, forming a “D” sign. The right ventricular end-diastolic diameter measured approximately 47.1  ×  69.9 mm (Figure 1). VA-ECMO was initiated. Before cannulation, a bolus of heparin at 50 U/kg was administered intravenously. Initial ECMO parameters were set at a rotational speed of 3,200 r/min, a flow of 3.5 L/min, and a gas flow rate of 4 L/min. Postcirculation, the activated clotting time (ACT) was 204 s, and the activated partial thromboplastin time (APTT) was 56 s. Given a strong suspicion of PE as the underlying cause, computed tomography pulmonary angiography (CTPA) was performed immediately, revealing multiple emboli in the main trunk of the right pulmonary artery and its branches (Figure 2A). The D-dimer level had risen to 68 mg/L. Considering the patient's stable vital signs under ECMO support, thrombolysis was deferred, and anticoagulation therapy with heparin was initiated, targeting an ACT of 180–200 s and an APTT of 60–80 s. On Day 3, a repeat CTPA showed partial resolution of the emboli in the right pulmonary artery and its branches (Figure 2B). A weaning trial on Day 4 was successful, and ECMO was discontinued. Post-decannulation, anticoagulation was maintained at the same ACT and APTT targets. By Day 13, CTPA demonstrated significant resolution of the emboli in the main trunk of the right pulmonary artery with good contrast filling (Figure 2C). On Day 23, the patient was successfully transferred out of the ICU and eventually made a full recovery. The hospitalization timeline and significant clinical events of this case are shown in Table 1.

**Figure 3:**
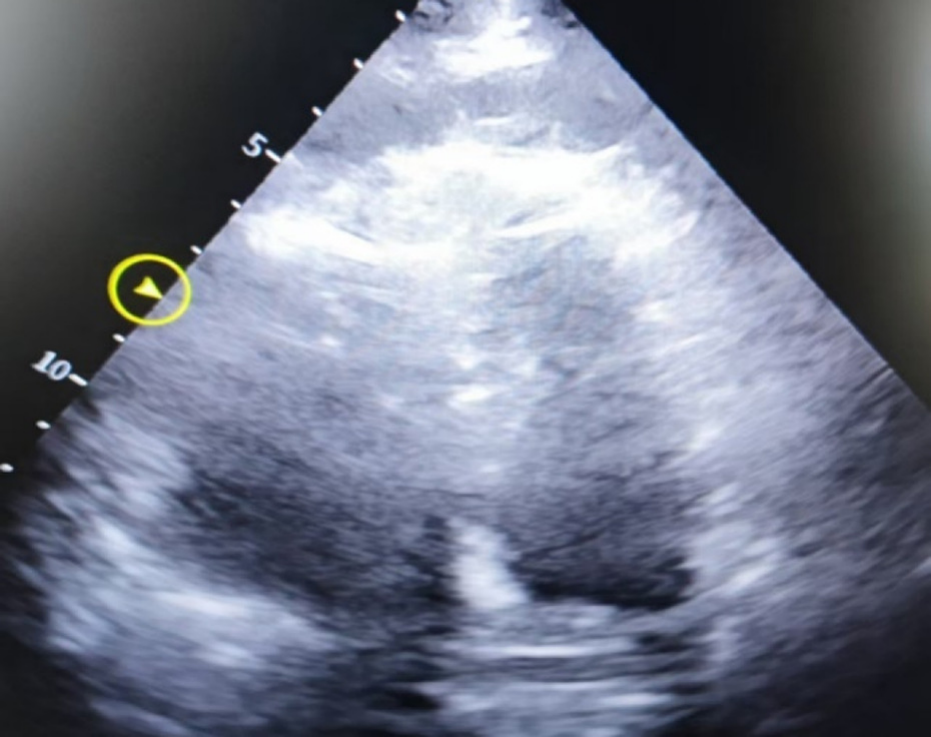
Apical four-chamber heart image, showing decreased left ventricular systolic function, severe dilatation of the right atrium and right ventricle, with the right ventricle measuring approximately 6.51 × 4.58 cm.

**Table 1:** Hospitalization timeline of the patient and the significant clinical events in Case 1.

Hospital day(s)	Timepoints	Significant clinical events
Day 0	10:00	The patient was treated at a local hospital because of symptoms of chest tightness and dizziness;
	15:00	The patient suddenly syncope and then had an in-hospital cardiac arrest, bedside CPR was immediately initiated;
	15:40	The patient temporarily recovered ROSC;
	15:50	The patient had cardiac arrest again, and bedside CPR was initiated immediately
	15:55	The patient recovered ROSC and relied on high-dose vasopressors to maintain vital signs;
	19:00	The ECMO team arrived at the local hospital and assessed the indications for ECMO;
	19:25	ECMO operation at 3,200r/min, Flow 3.5l/min, gas flow rate 4/min
	21:00	CTPA revealing multiple emboli in the main trunk of the right pulmonary artery and its branches
Day 3	10:00	CTPA showed partial resolution of the emboli in the right pulmonary artery and its branches
Day 13	10:00	CTPA demonstrated significant resolution of the emboli in the main trunk of the right pulmonary artery with good contrast filling

## Case 2

The patient is a 78-year-old elderly female who was admitted with a 2-year history of right lower limb pain and diagnosed with avascular necrosis of the right femoral head. A right hip arthroplasty was planned. She had a history of superficial venous thrombosis in both lower limbs and had been on long-term anticoagulation therapy with rivaroxaban, 10 mg once daily. The patient underwent total hip replacement surgery and was treated postoperatively with low-molecular-weight heparin (2,000 U subcutaneous injection) for anticoagulation on Day 1 after surgery. At 18:52, she experienced a sudden syncope after mobilizing, regaining consciousness after 2 min. At 19:21, she again became unresponsive, and CA was confirmed. Emergency tracheal intubation and CPR were initiated at the bedside. Resuscitation continued until 20:46 when ROSC was achieved. At that point, the circulation was maintained with adrenaline at 2.5 µg/kg/min and norepinephrine at 10.6 µg/kg/min. Cardiac ultrasound revealed decreased left ventricular systolic function and an enlarged right ventricle measuring approximately 6.51 × 4.58 cm, suggesting pulmonary embolism as the cause of the cardiac arrest. ECMO therapy was initiated without a heparin bolus prior to cannulation. By 21:22, ECMO was successfully established with initial settings of 2,800 rpm, flow 3.0 L/min, and gas flow 4 L/min. At 21:24, the patient was administered a 50 mg bolus of alteplase intravenously for thrombolytic therapy. At 22:45, the patient developed significant bleeding at the ECMO cannulation and central venous puncture sites, along with ECMO drainage line vibration and decreased ECMO flow. Laboratory testing revealed APTT > 180 s, PT > 120 s, and ACT > 999, indicating hemorrhagic shock and disseminated intravascular coagulation (DIC). Alteplase administration was immediately discontinued. Despite aggressive interventions, including transfusions of large volumes of fresh frozen plasma, red blood cells, and prothrombin complex concentrates, the hemorrhagic shock and DIC could not be reversed. ECMO failed to maintain adequate function, and the patient's vital signs deteriorated. Tragically, clinical death was pronounced on Day 2. The hospitalization timeline and significant clinical events of this case are shown in Table 2.

**Table 2:** Hospitalization timeline of the patient and the significant clinical events in Case 2.

Hospital day(s)	Timepoints	Significant clinical events
Day 0	12:00	Right hip replacement surgery.
Day 1	08:30	Subcutaneous injection of low-molecular-weight heparin 2,000 U for anticoagulant therapy
	18:52	The patient experienced sudden syncope after getting out of bed, with a heart rate of 98 bpm, blood pressure of 89/48 mmHg, and SPO_2_ of 85%
	18:54	The patient regained consciousness and complained of chest tightness and discomfort. At this time, the heart rate was 69 bpm, blood pressure was 117/55 mmHg, and SPO_2_ was 80%
	19:21	The patient experienced sudden respiratory and cardiac arrest, and cardiopulmonary resuscitation was initiated
	20:46	The patient has recovered from ROCS, but the blood pressure is very unstable, requiring high doses of vasoactive drugs to maintain blood pressure
	21:22	ECMO operation at 2,800 r/min, flow 3.0 L/min, and gas flow rate 4/min
	21:24	Administer 50 mg of alteplase intravenously for thrombolytic therapy
	22:45	The patient exhibited bleeding from multiple sites throughout the body, showing signs of DIC, accompanied by a rapid drop in blood pressure indicating hemorrhagic shock. The ECMO drainage tube was shaking, and thrombolysis was stopped
Day 2	15:51	Declared clinically dead

## Discussion

PE is a common and severe obstructive shock in clinical emergencies. It can lead to increased right ventricular afterload, potentially resulting in malignant arrhythmias and even CA. The mortality rate for patients with PE secondary to CA is as high as 84%–95% (2). According to the 2019 European Respiratory Society (ERS) guidelines, systemic thrombolysis is recommended as the first-line life-saving treatment strategy for patients with high-risk PE. For hemodynamically unstable high-risk PE patients, including those with CA or obstructive shock [systolic blood pressure <90 mmHg (1 mmHg = 0.133 kPa) or requiring vasopressors to maintain systolic pressure ≥90 mmHg despite adequate fluid resuscitation, along with signs of end-organ hypoperfusion] or persistent hypotension (systolic pressure <90 mmHg or a decrease of ≥40 mmHg lasting >15 min, excluding other causes such as new-onset arrhythmias, hypovolemia, or sepsis), mechanical circulatory support should be considered. VA-ECMO is an effective mechanical circulatory support option. ECMO provides near-total circulatory support, improves systemic perfusion, and offers additional time for PE diagnosis and treatment. However, the question of whether thrombolytic therapy is still necessary for patients with massive PE and CA treated with ECMO remains under investigation. At the very least, ECMO enables clinical decision-makers to deliberate more thoroughly on the optimal reperfusion strategy for PE—whether systemic thrombolysis, surgical embolectomy, or catheter-directed therapy. This allows for tailoring the clinical approach to minimize risks and maximize benefits based on the patient's specific etiology and clinical presentation (3).

Bleeding complications are among the most challenging issues for patients receiving ECMO support, and thrombolytic therapy significantly increases the risk of bleeding. Over 60% of ECMO-related events are associated with bleeding, which is closely linked to in-hospital mortality. In a study of 149 ECMO episodes in 147 adults, 89 episodes (60%) were complicated by at least one bleeding event. The most common bleeding sources included ECMO cannula sites (37%), hemothorax or cardiac tamponade (17%), and ear–nose–throat (ENT) bleeding (16%) (4). Another study found that over 30% of such patients required early termination of thrombolytic therapy and surgical exploration to manage bleeding. This rate is nearly three times higher than the bleeding risk associated with thrombolysis alone. The study reported that four out of 13 patients experienced severe bleeding complications, necessitating the premature cessation of thrombolysis followed by immediate surgical intervention. This included bleeding at cannulation sites in two patients and bleeding at previous abdominal and thoracic surgical sites in one patient (5). Maggio et al. (6) reported on 21 PE patients who underwent ECMO treatment, 56% of whom had contraindications to thrombolysis. Among these, neurological complications (such as intracranial hemorrhage) accounted for up to 50%, with four patients dying from cerebral hemorrhage. As a result, some experts consider thrombolytic therapy a relative contraindication during ECMO support due to the heightened risk of bleeding.

The 2018 European Guidelines on acute PE recommend pulmonary embolectomy as a viable treatment option for patients with life-threatening acute massive PE accompanied by shock, particularly in cases where thrombolysis is contraindicated or medical treatments such as thrombolysis have failed (3). Studies have also shown that the in-hospital survival rate for acute massive PE patients with cardiac arrest treated with ECMO can reach 61%. Additionally, the combination of ECMO and surgical embolectomy has been found to outperform ECMO combined with intravenous thrombolysis or ECMO alone (7). In a larger study of 53 PE patients treated with ECMO, the group that received thrombolysis before ECMO initiation (17 patients) had a mortality rate of 76.5%. Among these, 58.8% developed multiorgan failure. In contrast, patients undergoing pulmonary embolectomy with ECMO support had the greatest benefit, with a significantly lower mortality rate of 29.4% (8). During ECMO circulation, heparin can activate the body's endogenous fibrinolytic mechanisms, promoting thrombus dissolution while effectively preventing new thrombus formation. A small-sample study demonstrated that in six PE-induced cardiac arrest patients treated with ECMO, initial heparin therapy was administered via continuous intravenous infusion at 4–10 U/kg/h. The dosage was adjusted based on repeated monitoring of ACT, targeting a range of 180–220 s or an APTT 1.5–2.5 times the normal value. The study reported an 83.33% successful weaning rate from ECMO and a 30-day post-discharge survival rate of 66.67% (9).

## Conclusion

For patients with PE-induced cardiac arrest treated with ECMO, bleeding remains a major and uncontrollable complication of thrombolytic therapy. However, with advancements in extracorporeal life support technology, ECMO provides critical time for subsequent bridging treatments. This allows clinical decision-makers to carefully evaluate and choose the optimal treatment strategy.

## Data Availability

The raw data supporting the conclusions of this article will be made available by the authors, without undue reservation.
