# Correction: Case report: Does extracorporeal membrane oxygenation treatment for acute pulmonary embolism-induced respiratory and cardiac arrest still require thrombolysis?

**DOI:** 10.3389/fcvm.2025.1634497

**Published:** 2025-06-20

**Authors:** Fangfang Qiu, Bingxin Song, Lina Chen, Jiayi Hong

**Affiliations:** ^1^ Department of Critical Care Medicine, the Fourth Affiliated Hospital of School of Medicine, and International School of Medicine, International Institutes of Medicine, Zhejiang University, Yiwu, China; ^2^ Department of Respiratory and Critical Care Medicine, Center for Oncology Medicine, the Fourth Affiliated Hospital of School of Medicine and International School of Medicine, International Institutes of Medicine, Zhejiang University, Yiwu, China

**Keywords:** pulmonary embolism-diagnosis, extracorporeal membrane oxygenation (ECMO), thrombolysis, cardiac arrest (CA), disseminated intravascular coagulation (DIC)

**Correction on**
Case report: Does extracorporeal membrane oxygenation treatment for acute pulmonary embolism-induced respiratory and cardiac arrest still require thrombolysis? By Qiu F, Song B, Chen L and Hong J (2025). Front Cardiovasc Med. 12:1578970. doi: 10.3389/fcvm.2025.1578970

There was an error in Table 1 and its caption as published. The author mistakenly identified Table 2 as Table 1 which resulted in both table contents being similar. The corrected Table 1 and its caption appear below.

**Table 1:** Hospitalization timeline of the patient and the significant clinical events in Case 1.

Hospital day(s)	Timepoints	Significant clinical events
Day 0	10:00	The patient was treated at a local hospital because of symptoms of chest tightness and dizziness;
	15:00	The patient suddenly syncope and then had an in-hospital cardiac arrest, bedside CPR was immediately initiated;
	15:40	The patient temporarily recovered ROSC;
	15:50	The patient had cardiac arrest again, and bedside CPR was initiated immediately
	15:55	The patient recovered ROSC and relied on high-dose vasopressors to maintain vital signs;
	19:00	The ECMO team arrived at the local hospital and assessed the indications for ECMO;
	19:25	ECMO operation at 3,200r/min, Flow 3.5l/min, gas flow rate 4/min
	21:00	CTPA revealing multiple emboli in the main trunk of the right pulmonary artery and its branches
Day 3	10:00	CTPA showed partial resolution of the emboli in the right pulmonary artery and its branches
Day 13	10:00	CTPA demonstrated significant resolution of the emboli in the main trunk of the right pulmonary artery with good contrast filling

The original version of this article has been updated.

